# Polyol Process Coupled to Cold Plasma as a New and Efficient Nanohydride Processing Method: Nano-Ni_2_H as a Case Study

**DOI:** 10.3390/nano10010136

**Published:** 2020-01-12

**Authors:** Sonia Haj-Khlifa, Sophie Nowak, Patricia Beaunier, Patricia De Rango, Michaël Redolfi, Souad Ammar-Merah

**Affiliations:** 1 Université Paris 13, Sorbonne Paris Cité, CNRS UPR-3407, LSPM, 99 Avenue Jean-Baptiste Clément, 93430 Villetaneuse, France; soniahajkhlifa@gmail.com; 2 Université Paris Diderot, Sorbonne Paris Cité, CNRS UMR-7086, ITODYS, 15 rue Jean Antoine de Baïf, 75205 Paris, France; sophie.nowak@univ-paris-diderot.fr; 3 Sorbonne Université, CNRS UMR-7197, LRS, 2-4 Place Jussieu, 75005 Paris, France; patricia.beaunier@sorbonne-universite.fr; 4 Université de Grenoble Alpes, Grenoble INP, CNRS UPR-2940, Institut Néel, 25 Avenue des Martyrs, 38042 Grenoble, France; patricia.derango@neel.cnrs.fr

**Keywords:** nickel nanoparticles, polyol process, hydrogen cold plasma implantation, nickel hydrides, hydrogen storage

## Abstract

An alternative route for metal hydrogenation has been investigated: cold plasma hydrogen implantation on polyol-made transition metal nanoparticles. This treatment applied to a challenging system, Ni–H, induces a re-ordering of the metal lattice, and superstructure lines have been observed by both Bragg–Brentano and grazing incidence X-ray diffraction. The resulting intermetallic structure is similar to those obtained by very high-pressure hydrogenation of nickel and prompt us to suggest that plasma-based hydrogen implantation in nanometals is likely to generate unusual metal hydride, opening new opportunities in chemisorption hydrogen storage. Typically, almost isotropic in shape and about 30 nm sized hexagonal-packed Ni_2_H single crystals were produced starting from similarly sized cubic face-centred Ni polycrystals.

## 1. Introduction

The search for energy alternatives to reduce human dependency on fossil fuels has been a very pressing topic for the last few decades. Rapidly, hydrogen emerged as a carbon-free fuel with the highest known energy content, while emitting in its combustion, in a fuel cell or combustion chamber, only water as by-product [1]. This is particularly true when hydrogen gas is produced from water using renewable electricity (electrolysis) or solar energy (photoelectrolysis) and it is efficiently stored and transported. This closed cycle is expected to form the basis of a future CO_2_-free energy system, but only water-splitting hydrogen production is going to become an industrial reality [2,3]. Hydrogen storage is still technologically challenging. Indeed, hydrogen storage requires not only a high storage capacity but also good operability. Unfortunately, most high-density hydrogen storage media operate under particular conditions. For example, binding hydrogen to gases or liquids, converting it to other chemicals, like ammonia, methanol or formic acid, suffers from technical and economical limitations. For formic acid, a fueling infrastructure that can withstand the corrosive nature of this molecule is necessary [4,5,6]. For methanol, an additional chemical treatment is required to obtain a completely CO-free hydrogen [7]. Hydrogen physisorption on carbon materials [8,9,10,11] or on metal–organic frameworks [12,13,14], in which hydrogen molecules are held by means of weak van der Waals interactions with the substrates, requires an adsorption of hydrogen at relatively low temperatures, typically the temperature of liquid nitrogen, making hydride formation somewhat costly. Regarding hydrogen chemisorption on complex [15,16,17,18] or simple metal hydrides [19,20,21], in which atomic hydrogen is covalently bonded to the hosting crystal lattices, the exothermic character of the hydride formation reaction induces a heat release upon uptake of each mole of hydrogen. This heat must be removed during charging; otherwise, equilibrium temperature would be reached quickly and the reaction would stop. Conversely, to achieve a fast hydrogen release, it is necessary to supply the heat of the reaction, the desorption being an endothermic reaction. To achieve an efficient thermal management of a metal hydride tank, it is both necessary to improve the thermal conductivity of the metal hydride, e.g. with the introduction of expanded graphite [22] and to design a complex heat exchanger in the tank. Depending on the metal hydride nature, they can be ground to very fine particles with a high specific surface area [23,24] or directly produced as nanoparticles [25,26] to improve the sorption kinetics, but at the same time, inducing air and/or moisture sensitivity [27,28,29], requiring consequently a polymer coating to prevent the particle degradation [28,30,31,32,33]. 

Focusing on metal hydride nanoparticles, the achievement of superstoichiometries may compensate for the additional cost induced by coating, making these systems still economically and technically valuable. These superstoichiometries may be achieved only by ion implantation at adapted temperatures. In these conditions, a [H]/[Metal] saturation concentration ratio substantially higher that the limit observed in gas-phase charging can be reached. For instance, Myers et al. reached by ionic implantation of deuterium in palladium particles a saturation ratio as high as 1.6 ± 0.2 [34]. A superstructure was also observed in hydrogen-implanted Pd particles, reaching storage capacities similar to those obtained by very high-pressure hydrogenation of Pd [35]. Wulff et al., demonstrated that by ion implantation Pd hydrides can be formed during low temperature and low pressure plasma processing and that the resulting hydrides are stable at normal pressure and ambient temperatures [36].

All these preliminary results prompt us to suggest that plasma-based hydrogen implantation offer an alternative route for solid hydrogen storage and opens real perspectives since a large variety of finely divided metals could be used as starting materials. Of course, the added value, but also the difficulties of such a route, namely the hydrogen plasma interaction with metallic nanostructures, reside in the specific use of high-energy density processes, the control of which must allow either hydrogen implantation without severe damage (hydrogen-bubble, blistering … [37]). An alternative consists of performing hydrogen plasma surface interactions with granular nanometals using a high-frequency microwave generator. Such approach has already been successfully applied for hydrogen plasma interaction with structural materials relevant for fusion applications, like Al and W [38,39,40] for instance. In those cases, hydrogen bubbling was achieved and its effect on the mechanical properties of the treated metals was investigated. 

Within this general idea, we aimed to develop a new material-processing approach based on soft chemistry, namely the polyol process [41], and cold H_2_ plasma implantation, to produce non-usual metal hydride granular nanomaterials with a high and ideally reversible hydrogen storage capacity.

Therefore, to be as challenging as possible, we decided to apply this original hydrogenation route to a non-usual metal hydride, namely Ni metal. Usually, Ni requires ultra-high hydrogen gas pressure to form hydrides by classical hydrogenation route. According the Ni–H phase diagram established by Shizuku et al., stable hydrides NiH*x* can be formed up to *x* = 0.8 and temperatures *T* up to 800 °C, but only at high hydrogen pressures P ~1.1 and 5.4 GPa [42]. 

In this paper we present our main successful results on cold plasma hydrogenation of polyol-made Ni nanoparticles, as a compact of 13 mm in diameter and 1 mm in thickness, optimizing both plasma conditions and particle microstructure, highlighting the ability of this non-conventional route to easily produce the Ni_2_H phase. Preliminary works exist on hydrogen plasma implantation of Ni ultra-thin films [43], as a surface material processing, but to the best of our knowledge it was never investigated for volume material processing, underlining the importance of the nanostructuration of the starting metal.

## 2. Experimental Section

### 2.1. Materials 

Ni nanoparticles were produced by the well-known polyol process [41]. In practice, 2.2 g of tetrahydrated nickel acetate salt Ni(CH_3_CO_2_)_2_·4H_2_O (SIGMA-ALDRICH, Lyon, France ) and 4.4 g of polyvinylpyrrolidone (10^4^ g mol^−1^) abbreviated as PVP10000 (ACROS, Molinons, France) were dissolved in 125 mL of ethylene glycol OH(CH_2_)_2_OH abbreviated as EG (ACROS Molinons, France) and heated up to ebullition (180 °C with a step of 10 °C/min), in a glass flask under mechanical stirring. After 10 min of refluxing, 0.4 g of sodium borohydride NaBH_4_ (MERCK, Paris, France) were introduced in the reaction medium, and the mixture was then heated continuously for 2 h. The black suspension obtained was then recovered by centrifugation, alternating acetone washing (4 cycles) before to be dried in an oven (50 °C) for a couple of hours. The fresh powders were subsequently compacted (uniaxial pressure of 10 tons) as a pellet of 13 mm in diameter and 1 mm in thickness, which was then introduced in a plasma reactor equipped by a microwave generator (SAIREM, Décines-Charpieu, France) working at 2.45 GHz. The applicator used is a DP 10 model (BOREAL PLASMA, Le Pont-de-Claix, France). The microwaves are transmitted by a coaxial cable connected to a circulator with a water charge adapted to absorb the reflected power. The microwave power supplied was fixed to 180 W. More details about the reactors are given in Figure S1 in the supporting information section. The distance between the sample and the applicator was fixed at 2 cm, in order to optimize ions flow on Ni surface. The sample holder is cooled with water in order to limit the heat of the sample which may lead to hydrogen desorption from the sample. 

The pellet was then exposed for 6 h to a plasma gas mixture of H_2_/Ar (90/10) at a pressure of 1 mbar. Plasma treatment was carried out without polarization, assuming ion energy as proportional to that of plasma potential, i.e., 20 eV. In these conditions, hydrogen ions do not allow defect formation in Nickel, they can be only implanted [43] (see Table S1 in the supporting information section). The ionic current recovered at the sample holder is stabilized 5 min after the ignition of the plasma. Thus the current density crossing the Ni pellet surface is 5 mA cm^−2^ giving a hydrogen ions flux of 3.1 × 10^16^ ions.cm^−2^ s^−1^. For 6 h of plasma exposure, the associated hydrogen fluence becomes equal to 6.6 × 10^20^ ions cm^−2^.

### 2.2. Characterization

All the samples were powdered before characterization. Their crystalline structure was checked by X-ray diffraction (XRD) analysis using an X’pert-Pro diffractometer (Panalytical, Almelo, Netherlands), operating within the Bragg-Brentano θ-θ reflexion geometry, and equipped with a cobalt X-ray tube operating at 40 kV and 40 mA. The peaks indexing were performed with the program McMaille (version 3.02, Le Mans, France) [44]. The cell parameter and the size of the coherent diffraction domain were determined with MAUD software (version 2.55, Trento, Italy) [45] which is based on the Rietveld method combined with Fourier analysis, well adapted for broadened diffraction peaks. Polycrystalline strain free LaB_6_ certified by the National Institute of Standards was used as standard to quantify the instrumental broadening contribution. Finally, their detailed microstructural analysis was performed by transmission electron microscopy (TEM) using a JEM 2010 UHR microscope (JEOL, Tokyo, Japan), operating at 200 kV. In practice, small amounts of powdered nickel and nickel hydride were dispersed under ultrasonication in ethanol for a couple of minutes. A drop of each suspension was deposited on a 3 mm sized carbon coated copper TEM grids (EMS) and then dried in air. The images were collected with a 4008 × 2672 pixel CCD Gatan Orius SC1000 camera (AMETEK, Berwyn, IL, USA) and ImageJ software (2.0.0-rc-59, USA) was used to analyze them.

## 3. Results and Discussion

**Figure 1:**
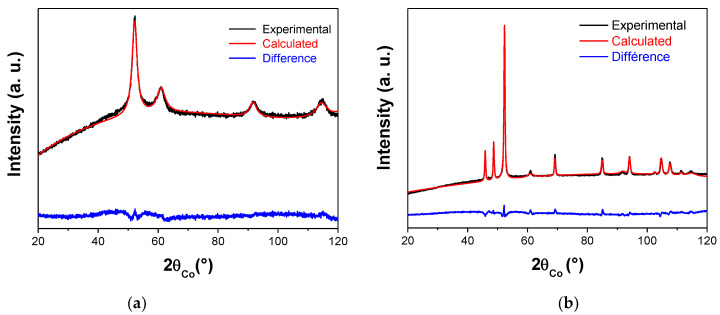
Experimental (black scatter) and calculated (red line) X-ray diffraction (XRD) patterns of polyol-made (**a**) and plasma treated (**b**) Ni powders. The residue, defined as the difference between the experimental and calculated diffractograms, is given (blue line) to illustrate the fit quality, with a Bragg reliability factor *R_B_* ranging between 1.5 and 2.0.

The structural analysis of both polyol-made and plasma-treated Ni powders was performed on fresh samples to be sure that the recorded patterns are representative of the as-produced systems. They were found to be matching very well with the face-centered cubic (fcc) Ni (ICDD n° 98-004-3397) and the hexagonal compact (hc) Ni_2_H (ICDD n° 98-020-1088) structures, respectively (Figure 1). Rietveld refinements confirmed that the former consisted of 100% fcc Ni nanocrystals while the latter was of 86% hc Ni_2_H nanocrystals and 14% fcc Ni ones (Table 1). 

Beside, focusing on the diffraction line broadening, the peaks of the hydride phase appear to be much narrower than those of the metal, suggesting a crystal size increase during the plasma treatment. Indeed, an average crystal size of about 4 nm was obtained for the Ni phase in the starting metallic powder while sizes of about 35 nm and 7 nm were, respectively, determined for the Ni_2_H and Ni phases, in the treated powder.

**Figure 2:**
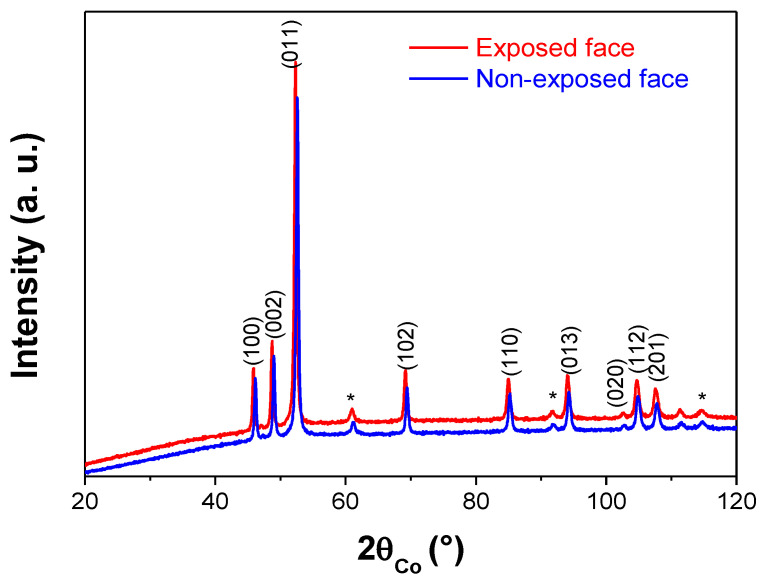
Experimental XRD patterns recorded on the directly exposed (red line) and non-exposed (blue line) to plasma implantation Ni pellet, within grazing conditions. Both were successfully indexed within the hexagonal Ni_2_H structure (ICDD n° 98-020-1088). The small peaks marked by an asterisk correspond to the diffraction planes of the cubic Ni structure (ICDD n° 98-004-3397).

The cold hydrogen plasma implantation experiment was repeated once to confirm the former results and the same XRD pattern was systematically obtained on the treated powder. Since plasma treatment proceeds usually on the surface of materials, we decided to repeat the experiment again and to maintain the sample in its pellet form to record its XRD pattern within grazing conditions, analyzing the two faces of the pellet, the exposed and the non-exposed ones. Once again the signatures of the Ni_2_H and Ni phases were identified in the same weight ratio than previously, for both faces, meaning that hydrogen ions diffused through the whole pellet thickness (Figure 2). These results point out the fact that the residual Ni contamination in the plasma treated sample is not due to an eventual lack of hydrogen diffusion across all the exposed material. 

Therefore, we decided to repeat the plasma implantation experiment shortening and prolonging the exposition time, down to 4 h and up to 8 h, supposing that the metal hydride kinetic requires longer operating time. For this new set of experiments we recorded again the same XRD patterns on the recovered pellets, with the same crystalline signatures within the same weight ratios (not shown).

**Figure 3:**
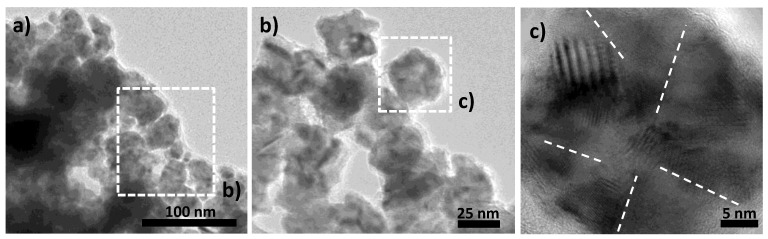
(**a**) Transmission electron microscopy (TEM) micrograph of the as-produced Ni powder. Only one population of particles can be identified: almost isotropic in shape polycrystals of 25–30 nm in size (**b**,**c**). More or less amorphous thin Ni hydroxide veils appear time to time around the Ni particles as a residual synthesis product (**b**). Note the white dashed lines are just guidelines.

As a first observation, the operating conditions cannot alone explain why all the starting metallic particles were not fully converted into their intermetallic counterparts during plasma exposition. Two reasons may be proposed: (1) a microstructural polydispersity of the starting particles making only some of them valuable for hydrogen implantation; and/or (2) a microstructural evolution, into the plasma reactor, making hydride transformation possible on fresh particles and not on aged ones. Transmission electron microscopy (TEM) micrographs were thus recorded on fresh metal powder to check its own microstructure. Representative images are given in Figure 3 at different magnitudes. They clearly show a homogeneous morphology. The powder consists of almost isotropic in shape particles of about 25–30 nm in size. These particles consist themselves of polycrystals, with a crystal size ranging between 4–8 nm. Looking attentively on the border of Ni polycrystals, a kind of an amorphous or poorly crystallized nickel rich matter appears. It was identified as a residual nickel hydroxide phase, since nickel production in polyol usually involves nickel hydroxide precipitation to serve as cation reservoir before Ni^2+^ reduction into Ni^0^ [41]. Its weak of crystallinity as well as its low content make this residual phase hard to detect by XRD. The presence of this additional phase was also confirmed by differential scanning calorimetry (DSC) and X-ray photoelectron spectroscopy (XPS) experiments. Indeed, about 200 mg of the as-produced Ni particles were introduced in a DSC capsule and heated up to 400 °C under argon. An endothermic peak very weak in intensity was observed at 360 °C, characteristic of nickel hydroxide reduction into nickel metal (Figure S2). Also, the Ni 2p XPS signal of the powder was recorded (Figure S3). This provided evidence of two series of 2p_3/2_ and 2p_1/2_ doublet: One, at respectively 852.8 and 870.0 eV, characteristic of nickel metal [46,47,48] and one, at respectively 855.8 eV and 873.9 eV, characteristic of nickel hydroxide [46,47,48].

**Figure 4:**
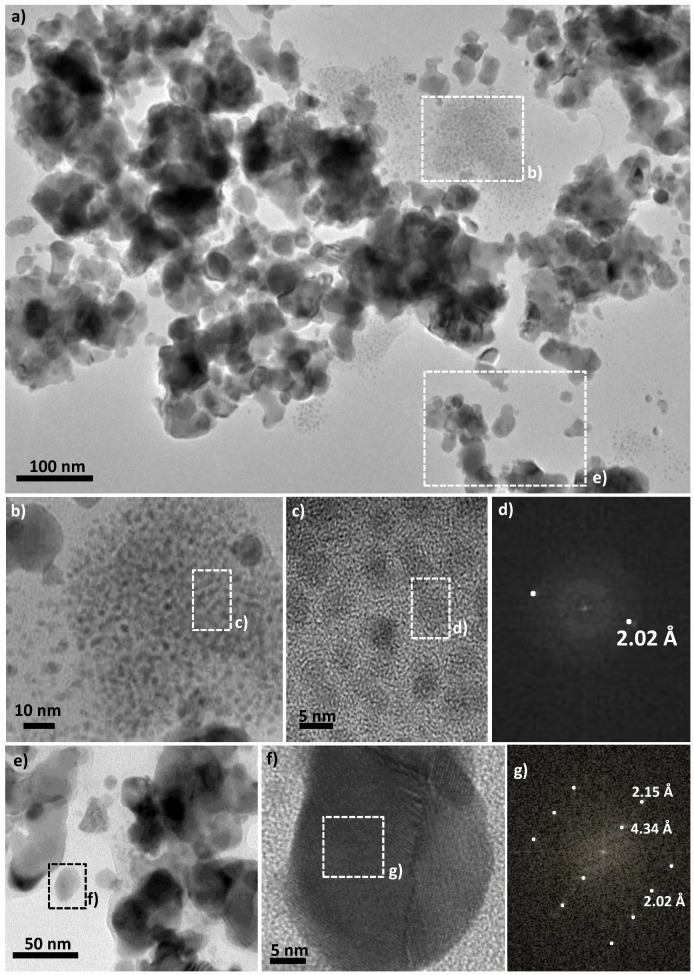
(**a**) TEM micrographs of the plasma treated Ni powder. Two populations of particles can be identified, the smallest and scarcest ones (**b**,**c**) and the largest ones (**e**,**f**). Fast Fourier transform (FFT) patterns calculated from high resolution TEM) images (**c**,**f**) recorded on representative particles of the two population are given and fully indexed within the fcc Ni structure (**d**) and the hc Ni_2_H one (**g**), respectively.

TEM micrographs were also collected at different magnitudes on the particles constituting the hydride powder to investigate their exact morphology. Interestingly, the observed microstructure is completely different from that described previously. Two populations of particles are present. The first, a majority, are large quite polydispersed single crystals, more or less agglomerated, as much as the minority, smaller, well dispersed and uniform in terms of microstructural morphology (Figure 4a). The latter is positioned on some ends of the larger particles, like previously, the residual thin nickel hydroxide veils on Ni particles before exposure to hydrogen plasma. We believe that, during plasma exposition, these veils are almost completely reduced into metallic particles, with an average diameter of 7 nm (Figure 4b,c). These small particles are almost isotropic in shape. Moreover, the fast Fourier transforms (FFT) calculated from the recorded High-resolution TEM (HRTEM) micrographs on some of them (Figure 4c,d) match very well with a Laue-type pattern indexed within the fcc Ni structure. Indeed only one distance, of 2.02 Å, is clearly identified. Moreover, even if this distance is common to the two Ni and Ni_2_H structures (the (111) and the (110), respectively), the scarceness of their related particles in the analyzed micrographs and the concordance of the TEM size of these particles with the XRD crystallographic coherent length of the metallic phase convinced us that this population is the metallic one. For unknown reasons, their formation stopped at the metallic stage and did not evolve to the intermetallic one. The largest particles are also single crystals with a size ranging between 25 to 45 nm, they are less rounded in shape (Figure 4e,f) and the FFT calculated from high-resolution TEM images recorded on representative particles (Figure 4g) fits very well with the hc Ni_2_H structure (ICDD n° 98-020-1088). The measured 4.34 Å (001) and 2.15 Å (002) distances on the Laue-type patterns are characteristic of the hexagonal lattice. The difference in size between these large particles and the previous small ones may explain why the hydride phase is stabilized within the former and not the latter. These results are very interesting because they highlight the effect of cold hydrogen plasma treatment on the metal to hydride reaction correlated with a poly- to a single-crystal microstructural transformation. 

They also point out the effect of the size of the starting metal (in situ or ex situ formed), since a minimal size, larger than 7 nm, is required to stabilize the intermetallic phase instead of the solid solution one. 

As a point of comparison, conventional H_2_ gas absorption experiments were performed on the as-produced Ni nanoparticles. In practice, the isotherm of H_2_ absorption was measured using a Sieverts’ type instrument, at room temperature, to be as close as possible to the operating conditions applied for hydrogen cold plasma treatment. Such a measurement is extremely important for conventional solid-gas hydrogen storage because it determines the hydrogen refilling capability. Interestingly, by increasing the pressure up to 70 bar, which is very high compared to the hydrogen pressure within our plasma reactor, the material does not uptake more than 100 µmol of H_2_ per 220 mg of powder, which is really negligible. Moreover, this hydrogen does not react with the metal to form its hydride counterpart, since the transformation plateau is not reached at all during these experiments (Figure S4). The recorded absorption curve was characteristic of reversible solid solution formation, far from any intermetallic storage phase stabilization. 

These results are very important because they provide clear evidence of the limit of the conventional material processing route compared to that described here, combining wet chemistry, namely the polyol process, to cold plasma treatment. Within relatively soft operating conditions almost pure granular Ni_2_H hydrides are produced reaching a hydrogen storage capacity of 0.9 wt.%. This is not negligible. As a point of comparison, this capacity does not exceed 1.4 wt.% and 6.0 wt.% in the state-of-the-art LaNi_5_H_6_ and MgH_2_ hydrides prepared by a conventional solid-gas hydrogenation process [49]. Of course, this experimental approach has to be optimized in terms of energy cost, material production and scale-up before being technically and economically considered for hydrogen solid storage applications. These preliminary results pave the way to explore a new material-processing route for metal hydride production and even though the question of the storage reversibility remains unaddressed to date, it is not so critical. Indeed, an alternative technological model can be imagined to solve it: a model based on the principle of returnable hydride disks. Plasma pre-charged hydride disks would be available in hydrogen city stations, and consumers would be able to exchange discharged disks by charged ones for their own hydrogen consumption (hydrogen fuel cell cars…).

## 4. Conclusions

As a proof of concept, we presented here our results on the synthesis by the polyol process of Ni nano- and polycrystalline particles, with an average size ranging between 25 and 30 nm, and their total transformation within Ni_2_H single crystals of almost the same size thanks to their treatment in a 2.45 GHz microwave plasma source at 180 W plasma power and 1 mbar working pressure and room temperature. operating temperature. We demonstrated that cold hydrogen plasma could be an efficient low-pressure and low-temperature transition metal hydrides bulk processing thanks to the nanostructuration of the starting granular materials. By this material processing route, a hydrogen storage capacity of 0.9 wt.% was reached, comparable to that reached by a conventional solid-gas hydrogenation process on the state-of-the-art LaNi_5_H_6_ hydrides, for instance.

## Figures and Tables

**Table 1:** Main structural and microstructural characteristics of the produced powders as inferred from XRD analysis. Typically the cell parameter *a*, the average size of the coherent diffraction domains <*L*> and the weight ratio of each identified crystalline phase are given.

	face Centered Cubic Structure: fcc-Ni			Compact Hexagonal Structure: hc-Ni_2_H		
	*a* (Å)	<*L*> (nm)	Ratio (wt.%)	*a and c* (Å)	<*L*> (nm)	Ratio (wt.%)
*Polyol-made Ni powder*	3.535(5)	4(1)	100	-	-	-
*Plasma-treated Ni powder*	3.535(5)	7(1)	14	2.651(5) 4.338(5)	35(5)	86

## Supplementary Materials

The following are available online at https://www.mdpi.com/2079-4991/10/1/136/s1.
