# Correction: Haj-Khlifa, S., et al. Polyol Process Coupled to Cold Plasma as a New and Efficient Nanohydride Processing Method: Nano-Ni_2_H as a Case Study. *Nanomaterials* 2020, *10*, 136

**DOI:** 10.3390/nano10040800

**Published:** 2020-04-21

**Authors:** Sonia Haj-Khlifa, Sophie Nowak, Patricia Beaunier, Patricia De Rango, Michaël Redolfi, Souad Ammar-Merah

**Affiliations:** 1 Université Paris 13, Sorbonne Paris Cité, CNRS UPR-3407, LSPM, 99 Avenue Jean-Baptiste Clément, 93430 Villetaneuse, France; soniahajkhlifa@gmail.com; 2 Université Paris Diderot, Sorbonne Paris Cité, CNRS UMR-7086, ITODYS, 15 rue Jean Antoine de Baïf, 75205 Paris, France; sophie.nowak@univ-paris-diderot.fr; 3 Sorbonne Université, CNRS UMR-7197, LRS, 2-4 Place Jussieu, 75005 Paris, France; patricia.beaunier@sorbonne-universite.fr; 4 Université de Grenoble Alpes, Grenoble INP, CNRS UPR-2940, Institut Néel, 25 Avenue des Martyrs, 38042 Grenoble, France; patricia.derango@neel.cnrs.fr

The authors wish to make the following corrections to this paper [1]: there are two mistakes in this article [1]. In the last paragraph of Section 3—Results and Discussion, the sentence “Within relatively soft operating conditions almost pure granular Ni_2_H hydrides are produced reaching a hydrogen storage capacity of 1.7 wt %” should be “Within relatively soft operating conditions almost pure granular Ni_2_H hydrides are produced reaching a hydrogen storage capacity of 0.9 wt %”. In Section 4—Conclusions, the sentence “By this material processing route, a hydrogen storage capacity of 1.7 wt % was reached” should be “By this material processing route, a hydrogen storage capacity of 0.9 wt % was reached”.

The authors would like to apologize for any inconvenience caused to the readers by these changes.
