# Supplementary material for: Polyol Process Coupled to Cold Plasma as a New and Efficient Nanohydride Processing Method: Nano-Ni2H as a Case Study
Source: Nanomaterials (Basel). 2020 Jan 12;10(1):136. doi: 10.3390/nano10010136 (PMC7022929; doi:10.3390/nano10010136)
Supplement: Supplementary file 1 [file nanomaterials-10-00136-s001.pdf]

Polyol process coupled to cold Plasma as a new and efficient nanohydride processing: nano-Ni_2H as a case study

Sonia Haj-Khlifa [1,2], Sophie Nowak [2], Patricia Beaunier [3], Patricia De Rango [4], Michaël Redolfi [1,*] and Souad Ammar-Merah [2,*]

[1] Université Paris 13, Sorbonne Paris Cité, CNRS UPR-3407, LSPM, 99 Avenue Jean-Baptiste Clément, 93430 Villetaneuse, France
[2] Université Paris Diderot, Sorbonne Paris Cité, ITODYS, UMR 7086, CNRS, 15 rue Jean Antoine de Baïf, 75205 Paris, France
[3] Sorbonne Université, CNRS UMR-7197, LRS, 2-4 Place Jussieu, 75005 Paris, France
[4] Université de Grenoble Alpes, CNRS, Grenoble INP, Institut Néel, 38000 Grenoble, France

Supporting information

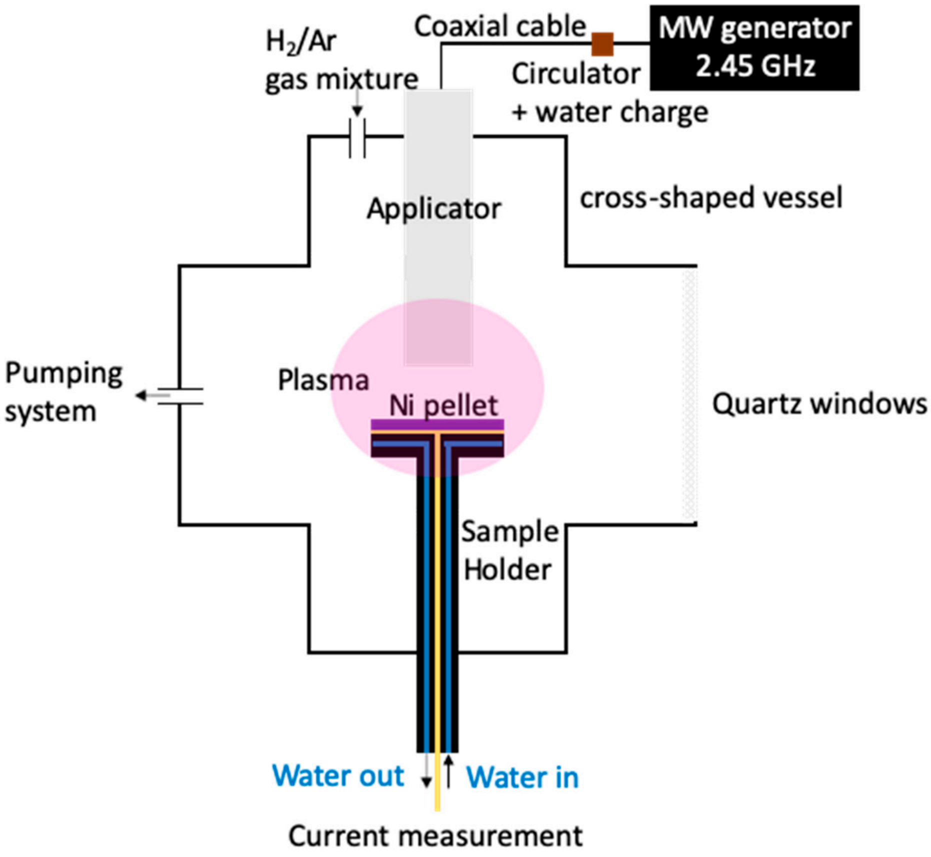

Figure SI. Scheme of the plasma reactor. It consists of a cross-shaped stainless steel vessel with a total volume of 5 dm^3. It has 6 ports or openings (DN 100) which have multiple uses: pumping system, sample holder, plasma source and plasma diagnostics through quartz windows. The system is composed of two pumps: a primary dry pump (Alcatel ACPA15) and a turbo-molecular pump (Pfeiffer) with a pumping rate of 250 $L.s^{-1}$ that allows reaching a vacuum limit of 10^{-6} mbar. The pressure is measured by an MKS capacitive gauge (range 1 mbar). The reactor has a manual valve in front of the pumping system that allows isolating the reactor when needed. The gas flow is controlled by Bronkhorst flowmeters. Each

flowmeter is calibrated for the gas it controls (from 0 to 100 sccm). A schematic representation of the complete plasma implantation setup is given hereafter.

	$E_{defects}$ (eV)
Ni/H^+	519
Ni/H_3^+	185
Ni/Ar^+	36

Table SI. Threshold energy of defects formation in Ni/H^+, Ni/H_2^+ and Ni/Ar^+ interactions deduced from $E_{defects} = \frac{(M_{ion}+M_{Ni})^2 \times E_{displacment}}{4M_{ion} \times M_{Ni}}$, which traduces the fact that defect creation energy depends on the mass of ion and nickel and the atom displacement energy (i.e. vacancy production) in nickel. According our operating conditions, only heavy argon ion Ar^+ can create defects inside the material at low energy unlike hydrogen ions. Vacancies creation allows having new storage sites in addition to interstitials sites.

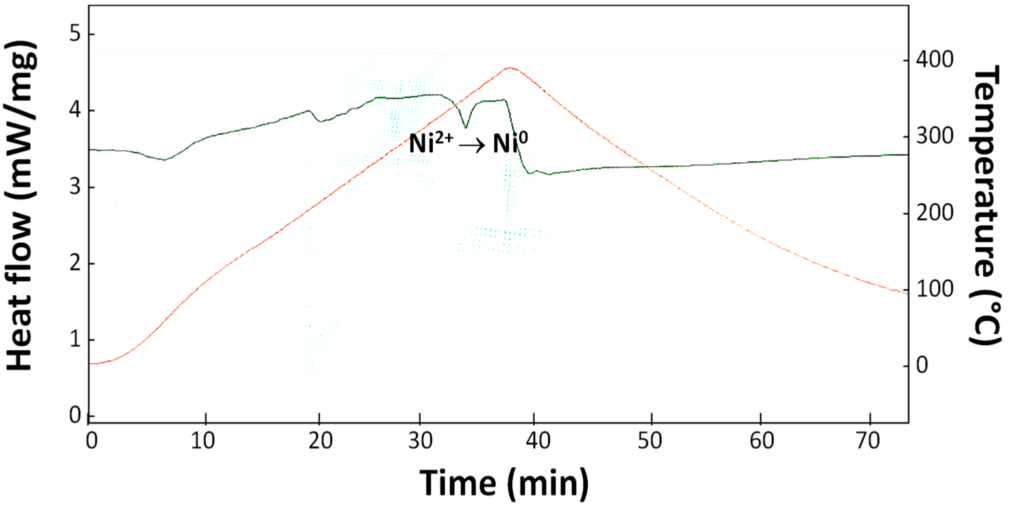

Figure S2. DSC plots recorded on the as-produced Ni powder heated under Argon up to 400°C (10°C/min). A Thermo Fisher Scientific machine was used for the measurement.

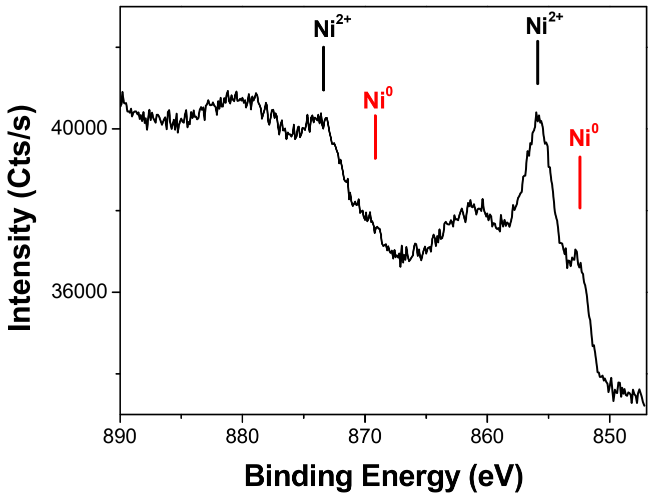

Figure S3. Ni 2p X-ray photoelectron spectra (XPS) recorded on the as-produced Ni powder on a Thermo VG ESCALAB 250 instrument equipped with a micro-focused, monochromatic A1 Kα X-ray source (1.486.6 eV). A magnetic lens was used while fixing the X-ray spot size to 500 µm (15 kV, 150 W). All the spectra were acquired in the constant analyzer energy mode with a pass-energy of 40 eV.

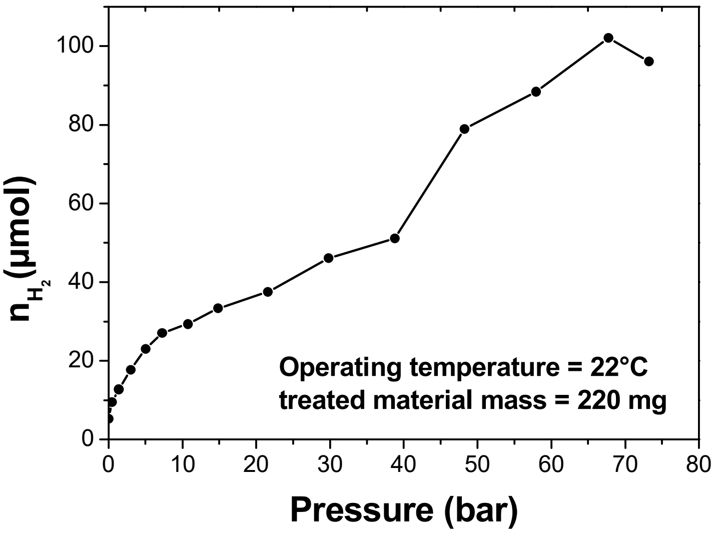

Figure S4. Isothermal hydrogen absorption curve recorded at r.t. thanks to a Sieverts' type instrument on the as-produced Ni particles, using a Pressure-Composition isotherm measurement device (IMI-PCT) purchased from Hiden Isochema (model HTP1-S).
